# Neural control of submucosal gland and apical membrane secretions in airways

**DOI:** 10.14814/phy2.12398

**Published:** 2015-06-09

**Authors:** Alan W Cuthbert, Meena Murthy, Alexander P S Darlington

**Affiliations:** Department of Medicine, University of CambridgeCambridge, UK

**Keywords:** Submucosal gland secretion, transepithelial transport, veratrine alkaloids

## Abstract

The mechanisms that lay behind the low-level secretions from airway submucosal glands and the surface epithelium in the absence of external innervation have been investigated in small areas (1.0–1.5 cm^2^) of mucosa from sheep tracheas, freshly collected from a local abattoir. Glandular secretion was measured by an optical method while short circuit current was used as a measure of surface secretion. Activation of neurones in the intrinsic nerve net by veratrine alkaloids caused an immediate increase in both glandular secretion and short circuit current, both effects being blocked by the addition of tetrodotoxin. However, agents known to be acting directly on the glands, such as muscarinic agonists (e.g., carbachol) or adenylate cyclase activators (e.g., forskolin) were not influenced by tetrodotoxin. The toxin alone had no discernable effect on the low-level basal secretion shown by unstimulated glands. Calu-3 cell monolayers, generally agreed to be a surrogate for the secretory cells of submucosal glands, showed no sensitivity to veratrine alkaloids, strengthening the view that the veratrine-like drugs acted exclusively on the intrinsic nerve net. The data are discussed in relation way in which transplanted lungs can maintain mucociliary clearance and hence a sterile environment in the absence of external innervation, as in transplanted lungs.

## Introduction

In the insightful review by Wine (2007) it is postulated that antimicrobial secretions of mucus from airway submucosal glands can be independent of centrally mediated airway defense reflexes and that an innate airway defense system comprising of low level gland secretion is sufficient to maintain mucociliary clearance. Such an in-built system would explain how transplanted lungs maintain patency and sterility over long periods in the absence of external innervation. It is postulated by Wine that noxious stimuli, such as air-borne pollutants including air-borne bacteria, viruses, and spores activate sensory nerve endings of intrinsic neurones, which in turn affect glands by local reflexes to secrete antimicrobial-rich mucus, that together with a smaller contribution from surface secretion, is able to maintain efficient mucociliary clearance, keeping the airways clean and free from infection. This study is designed to test the Wine postulate.

It is well known that the enteric nervous system allows the gut to show reflex activity in the absence of input from the brain or spinal cord. While far less well developed in airways it is suggested that neural elements of the mucosal plexus of the major airways form an airway intrinsic nervous system that can help control gland secretion, blood flow, and muscle tone. Limiting our investigations to the control of secretion we have looked for evidence that the innate nervous system in the sheep trachea can influence gland secretion and mucosal surface secretion.

To gain an understanding of the neuroanatomy in the airways of a variety of species readers should consult the review referred to above (Wine 2007). No study has reported the innervation of an individual gland cell or an individual surface epithelial cell, since in the ANS no specialized neuroffector apparatus is found, rather neurones terminate in the region of the effector cells so that the response of a single cell may derive from the input of more than one nerve terminal or alternatively be too distant from any currently active terminal to show any response (Burnstock 1988). Some VIP-containing neurones have been shown to send projections along the basolateral aspects of the epithelium (Dey et al. 1999) but without forming structural synapses.

Our major aim in this study was to obtain definitive evidence that the secretory activity of airway submucosal glands and the airway surface epithelium can be activated by neural activity generated in the local innate nerve net.

Most of the data reported in this study were obtained using small pieces of ovine mucosa, freed of muscle and cartilage, dissected from the ventral surface of the trachea, and, containing part of the mucosal nerve plexus. Wine (2007) has summarized the evidence that the neural networks in the trachea form functional networks and from the massive amount of pharmacological work done has shown that substance P and vasoactive polypeptide are likely to be the major transmitters involved (Widdicombe 2010).

Our initial approach was to apply electrical field stimulation to activate network neurones but no convincing TTX-sensitive responses were recorded. This may reflect the paucity of the innervation compared to the intestine where electrical field stimulation readily gives myogenic and secretory responses. Indeed we only found one example in airways (Ianowski et al. 2007) where electrical stimulation resulted in glandular secretion. This was found in the mouse and where the whole trachea was placed in the recording chamber with stimulating electrodes at both ends of the preparation. It seems unlikely in this circumstance that the stimulation is confined to the mucosal nerve plexus. Thus, attention was given to chemical methods of activating cells with excitable membranes, such as those found in neurones. The veratrine alkaloids are known to be activators of voltage-sensitive sodium channels as found in neurones and skeletal muscle. The specificity of this approach can be easily tested, for example, by using a high-affinity blocker of voltage-sensitive sodium channels. Tetrodotoxin (TTX) is such a compound and should completely remove all and any effects caused by the alkaloids. There are nine classes of voltage gated sodium channels known as Na_v_1.1 to Na_v_1.9. All except Na_v_1.8 and Na_v_1.9 are known to be activated by veratrine alkaloids, while all nine are blocked by TTX (Alexander et al. 2011).

Our initial objective was to examine if activation of the mucosal plexus caused gland secretion or altered the secretory current across the short circuited epithelial mucosa. A secondary objective was to explore further the origin of the so-called surface secretion, generally considered to be unassociated with submucosal gland function.

## Materials and Methods

### Solutions

Krebs–Henseleit Solution (KHS) was used throughout for bathing tissues. Its composition was (mmol/L): 117 NaCl, 4.7 KCl, 2.5 CaCl_2_, 1.2 MgSO_4_, 1.2 KH_2_PO_4_, 25 NaHCO_3_, and 11.1 glucose. The pH was 7.4 after bubbling with 95% O_2_/5% CO_2_.

### Tissues

Most experiments were made using tissue obtained from sheep tracheas obtained from freshly killed animals at a registered abattoir. In a few other experiments cultured monolayers of Calu-3 cells were used as a test tissue and were cultured in the laboratory.

### Methods

#### Measurement of secretion from individual tracheal submucosal glands

Experiments reported here were made with pieces of airway mucosa dissected from the ventral surface of sheep tracheas. Ovine tracheas were collected from the abattoir into chilled and oxygenated Krebs–Henseleit Solution (KHS) and generally used within 8 h. Small pieces of mucosa (1–1.5 cm^2^) were dissected from the ventral airway surface and mounted in such a way that the serosal side could be bathed in oxygenated KHS maintained at 37°C while the apical surface, after cleaning and drying, was covered with a thin layer of water saturated mineral oil. Secretions from individual glands formed spherical bubbles under the surface of the oil. The surface of the tissue was photographed at intervals throughout the experiment and stored. The captured images were calibrated by inclusion of a 0.5 mm grid placed upon the epithelial surface at the start of the experiment. Agents could be added to or removed from the serosal surface by changing the solution bathing the basolateral side of the mucosa. When it was necessary to apply an agent to the apical side it was a requirement that the agent could dissolve in the oil used to cover the mucosal surface. Small volumes (1 *μ*L) of this solution could be added to the surface and allowed to diffuse toward the tissue. In all the experiments in which secretion from individual glands was measured the n-values indicate the number of glands that were successfully monitored for the duration of the experiment from a single tissue derived from a single animal. In all a total of 13 tracheas were used to carry out the experiments reported in this study.

Using Image J software (NIH, USA) the bubble sizes were converted to volumes and secretion rates after the experiment was completed. Only mucus bubbles that retained their circular outline and did not coalesce with adjacent bubbles were used in the analysis. Full details of the methodology are given elsewhere (Joo et al. 2001, 2009; Wine et al. 2011). Quite frequently tiny bubbles not associated with submucosal glands can be seen under the oil that maybe due to the outward movement of tiny amounts of aqueous fluid across the surface epithelium. These tiny bubbles are often referred to as surface secretion.

### Short circuit current recording

#### Short circuit current recording (SCC) from isolated sheep tracheal mucosa

As above observations were made from small pieces of mucosa dissected from the ventral surface of the trachea. They were mounted in Ussing chambers with a window area of 0.64 cm^2^. Nonpolarizable voltage sensing electrodes were introduced into ports in each Ussing half chamber close to the epithelial surface, while current-passing electrodes were inserted into ports in each half chamber away from the epithelial surface. Both sides of the tissue were bathed in KHS which was maintained at 37°C and circulated by a gas lift powered by the 95% O_2_/5% CO_2_ supply. Tissues could be voltage clamped at zero potential using a WPD Dual Voltage clamp-1000. Fluid resistance compensation was used throughout and SCC was recorded continuously during the experiment using an ADInstruments Powerlab 8SP and displayed on screen. Further details of short circuit recording from sheep tracheas can be found in earlier papers (Joo et al. 2009).

### SCC recording from Calu-3 cell monolayers

Calu-3 cells were grown in 75 cm^2^ culture flasks containing Eagle's Minimum Essential Medium with 10% fetal calf serum, penicillin G, 6 *μ*g/mL, and streptomycin, 10 *μ*g/mL. Cells were incubated at 37°C in humidified air containing 5% CO_2_. Cells from confluent monolayers were collected by trypsinisation and subcultured on Snapwell polycarbonate membrane inserts (Costar UK) (1 cm^2^ 0.4 *μ*m pore size). Cultures on membrane inserts were refed every 3–4 days and used at around 4 weeks. The detachable rings of the Snapwell inserts, bearing the cultured monolayers, were inserted into CHM5 Ussing chambers with associated electrodes and voltage clamped as described above for isolated tracheal mucosa. Further details can be found in Cuthbert et al. (2003).

### Reagents

The following reagents were obtained from Sigma-Aldrich (Gillingham, UK); amiloride, atropine, bumetanide, capsaicin, carbachol, forskolin, veratrine, veratridine.

Tetrodotoxin came from Tocris Bioscience and 4-chlorobenzo(F)isoquinoline from Ubichem plc. Note veratrine is predominantly veratridine together with other similarly acting alkaloids of natural origin.

## Results

To assess whether stimulation of neurones within the innate nerve plexus of the tracheal mucosa can lead to secretion of mucus from the submucosal glands, we chose to use agents that specifically and maybe exclusively, stimulate voltage-sensitive sodium channels, and hence activate conducted action potentials in nerves. Veratrum alkaloids are considered to have these properties (Ulbricht 1969; Barnes and Hille 1988).

**Figure 1:**
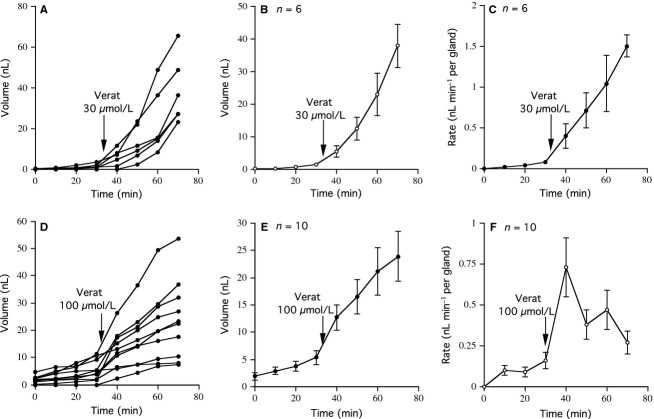
Submucosal gland secretion in response to veratrine (Verat). The figure shows data from two experiments in which airway submucosal gland secretion was measured in response to veratrine. In the first (A to C) secretion volume versus time is shown for six separate glands (A), with the mean values shown in (B), while the mean secretion rate for all six glands, as nL/min per gland is shown in (C). The format of the second experiment is identical, as shown in (D), (E), and (F), but in this instance the veratrine concentration was increased and data from 10 separate glands are given.

The data in Fig.1 indicate some of the basic features of veratrum alkaloid-stimulated secretion of submucosal gland mucus measured using small (circa 1 cm^2^) areas of mucosa dissected from ovine tracheas. In the first experiment (Fig.1A, B, and C), addition of veratrine, 30 *μ*mol/L to the basolateral face of the mucosa produced an immediate increase in the volume of secretion-associated with six individual glands (Fig.1A), while Fig.1B shows that the mean value of the secreted volume gives a similar pattern to that of individual glands. The increased volumes of secretion reflect an increase in secretion rate as shown in Fig.1C. When the experiment was terminated neither the mean secreted volume nor the mean secretion rate had apparently reached peak values, although the mean secretion rate had increased some 15 times. In a second experiment (Fig.1D, E, and F), the fortunes of 10 separate glands in a tissue from a different animal were followed during 70 min. The data here are similar to the first experiment, except that when the experiment was terminated clearly the maximal rate of secretion had passed. The reasons why the responses in these two examples are not ‘dose related’ is unknown, but dissection and preparation of the tissues for experimental recording is likely to have damaged many local neurones in the submucosa. Also the distribution of the innervation is unlikely to be equal in different samples of tissue from different animals, furthermore time since culling, age of the animal, and sizes of the individual glands are likely contributors.

**Figure 2:**
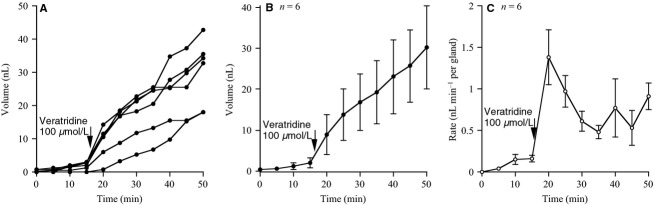
Submucosal gland secretion in response to veratridine. The figure shows the secretion volumes versus time for six separate glands (A), the mean secretion volumes for all glands (B), and the mean secretion rates for all glands (C).

Figure2A, B, and C shows data from an experiment in which veratridine was used to stimulate submucosal gland secretion. The results again take the form found with veratrine and in no experimental condition did we discover characteristics that could differentiate veratrine from veratridine.

**Figure 3:**
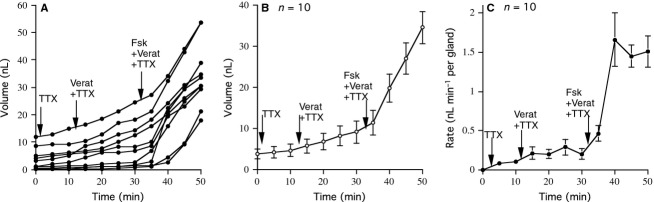
Effect of tetrodotoxin (TTX) on the responses to veratrine on submucosal gland secretion. The figure shows the volume of secretion from 10 separate glands versus time (A) and the mean secretion volumes for all 10 glands (B) and the mean secretion rates for 10 glands (C). At the start of the experiment TTX (1 *μ*mol/L) was present in the basolateral fluid to which was added veratrine (100 *μ*mol/L) at 15 min and further supplemented by forskolin (Fsk), 10 *μ*mol/L at 35 min.

Tetrodotoxin is known to be a specific blocker of voltage-sensitive sodium channels (Kao 1966) and therefore expected to block conducted action potentials and hence the effect of the veratrine alkaloids. When TTX was present from the beginning of the experiment addition of veratrine produced no change in the rate of secretion, while subsequent addition of forskolin, acting directly on the glands and bypassing any neural involvement gave a rapid change in the mean secretory volume and secretion rate, conforming to the requirement of the model outlined above (Fig.3A, B, and C).

**Figure 4:**
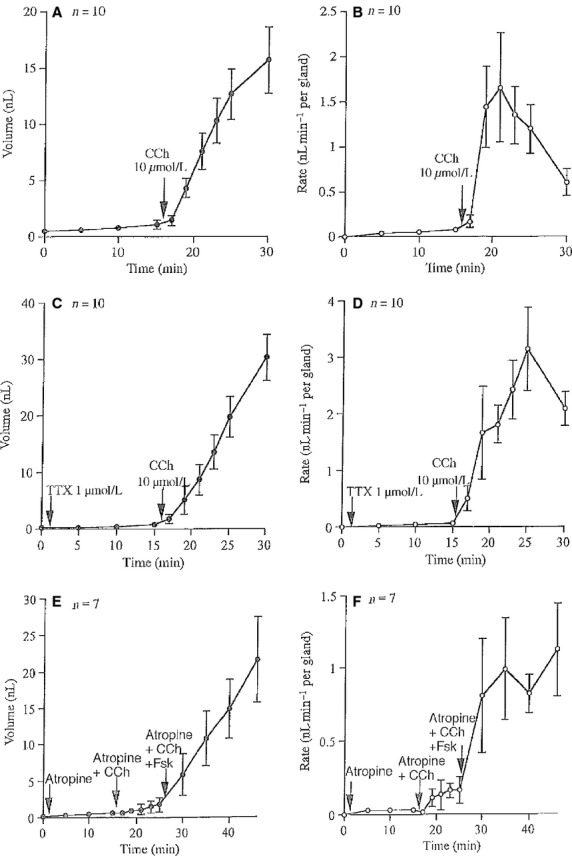
Illustrates the effects of carbachol (CCh) on tracheal secretion in three separate experiments. In the first (A and B) typical responses to carbachol (CCh) for 10 glands are shown. The second experiment (C and D) shows the responses to carbachol are not prevented by TTX, again for measurements from 10 glands. The third experiment (E and F) with measurement from seven glands shows that atropine, 0.1 *μ*mol/L almost completely blocks the response to to CCh, 10 *μ*mol/L but does not prevent responses to forskolin, 10 *μ*mol/L. In each experiment, mean secretory volumes and mean secretory rates are given.

A further example that differentiates effectors acting directly on the glands to produce secretion rather than indirectly through neural innervation can be shown with muscarinic agonists, such as carbachol, considered to act upon M3 receptors on the gland surface (Fig.4A and B) (Joo et al. 2001). This effect of carbachol is repeatable in the presence of TTX, sufficient to inhibit completely any neural transmission as indicated in Fig.4C and D.

Furthermore, if a low concentration of a muscarinic antagonist, such as atropine (100 nmol/L) is used to inhibit carbachol-induced secretion, forskolin is still able to stimulate secretion by direct action as can be deduced from the data of (Fig.4 E and F).

We conclude from these results that veratrine and forskolin (or carbachol) act independently with different modes of action to cause mucosal gland secretion. Therefore, it was not surprising that the effects of veratrine followed by forskolin were found to be additive measured as secretion volume or secretion rate (data not shown).

**Figure 5:**
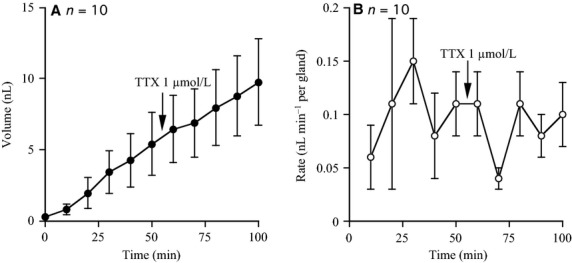
Long-term effects of tetrodotoxin (TTX, 1 *μ*mol/L) on airway submucosal gland basal secretion. The volume of secretion from 10 glands was measured every 10 min for 100 min. TTX, 1 *μ*mol/L was added after 50 min. Mean secretory volumes and mean secretory rates for 10 glands are shown in (A) and (B).

If low level neural activity was a component of basal, innate glandular secretion process we would expect that TTX alone would affect the basal rate of secretion. Basal secretion before and after the addition of TTX was measured, each for a 50 min period. Figure5A and B shows the result of measurements from 10 glands. No evidence was found that TTX influenced the basal secretory rate that remained at around 0.1 nL/min per gland after toxin addition. A similar observation can be made from the data at the start of Figs.3, 4C and D and has also been commented on by others (Joo et al. 2001).

Not all of the innervation that serves the ovine tracheal mucosa is directed to the submucosal glands and some fibers may well terminate close to the surface epithelium, a structure that is capable of secreting salts and water, thus adding to the total volume of airway secretion. Since there are no structural neuroeffector junctions in the autonomic system (Burnstock 1988) it cannot be inferred that neural effects can influence surface secretion.

The contribution to transport by the surface epithelium can be assessed by measurement of SCC, keeping in mind that transport of salts in either direction across an epithelium requires equivalent water movement to maintain osmotic equilibrium.

**Figure 6:**
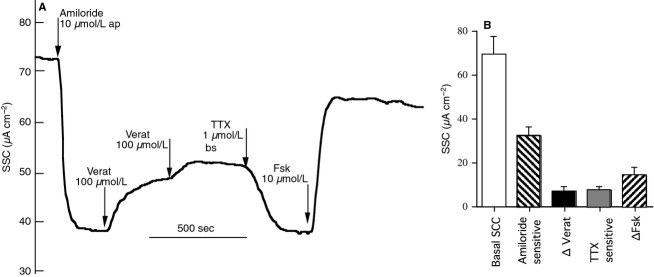
Shows the effect of veratrine on SCC of ovine tracheal mucosa. Veratrine applied to either side of the mucosa caused an increase in SCC in the presence of amiloride to inhibit electrogenic sodium transport. The increase was rapidly blocked by addition of TTX, 1 *μ*mol/L, while the current could be further increased by forskolin (Fsk) (A). Data from five identical experiments are given in (B). The current added by veratrine addition was not significantly different from that removed by TTX.

Figure6A and B shows results from a series of experiments in which veratrine was able to increase SCC in the presence of amiloride at a concentration sufficient to completely block sodium absorption, suggesting the responses resulted from anion secretion that was rapidly reversed by TTX, while allowing a further current increase following addition of forskolin. The commonalities between these results and those found with glandular secretion are obvious. Note too that veratrine can be active from either the apical or the basolateral side of the tissue, probably a function of its high lipid solubility. Similar results to these were also obtained with veratridine. In further experiments (data not shown), the SCC increase due to either veratrine or veratridine showed no sensitivity to atropine. This makes it unlikely that parasympathetic (cholinergic) fibres involved in acute airway defense mechanisms play a significant part in these observations. The cellular events associated with these changes in SCC must also have occurred when we recorded in the glandular secretion mode and raises the question how the effect on surface secretion is expressed when glandular secretion is being recorded. One possibility is so called ‘surface secretion’ to describe the accumulation of tiny bubbles of secretion occurring at a density far >1/mm^2^, the normally accepted density for submucosal glands. If this is so then an increase in neural activity should affect the surface secretion at the same time as glandular secretion is increased.

**Figure 7:**
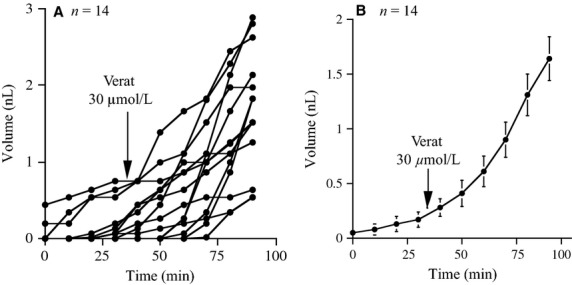
Effect of veratrine on (so called) surface secretion. The fate of 14 small bubbles (<1 nL) was followed for more than 1 h following addition of veratrine 30 *μ*mol/L. Individual and mean volumes are given.

We found that the average growth rate of the bubbles increased from 4.3 pL/min to 34 pL/min when veratrine was added (Fig.7A and B). It should be remembered that the effects on SCC are rather modest and therefore the volume of surface secretion added will be small compared to the volume contributed by the glands (Trout et al. 2001).

It has been shown that the noxious stimulus capsaicin applied apically causes CFTR-dependent secretion in porcine and human tracheal submucosal glands that was partially inhibited by TTX (Khansaheb et al. 2011) indicating neural involvement. In these experiments, capsaicin was dissolved in the same oil used to cover the mucosal surface and small volumes were added to the surface and diffusion allowed to occur.

**Figure 8:**
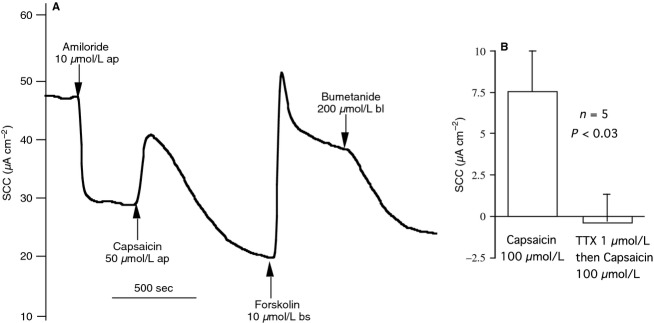
A illustrates the effect of capsaicin, applied apically(ap) to the short circuited tracheal mucosa in the presence of amiloride, followed by forskolin and bumetanide applied either basolaterally(bl) or both sides (bs) of the epithelium (A). (B) shows the effect of TTX on the responses to capsaicin on SCC.

Many attempts to replicate the earlier findings with pig and human tissues in sheep tissue were unsuccessful. However, when ovine tracheal epithelia were used in the SCC measuring mode apical application of capsaicin caused an immediate increase in SCC (Fig.8A). SCC responses to capsaicin rapidly ‘desensitised’ so that after 5–10 min the currents fell to values lower than that obtaining before the capsaicin was added. Attempts to obtain a concentration response curve to capsaicin were frustrated since addition of increasing concentrations gave ever decreasing responses as desensitisation to the earlier exposures took hold. Importantly, in the presence of TTX no responses to capsaicin on SCC were seen (Fig.8B), suggesting the responses were neurally mediated, presumably by neurones from the mucosal plexus.

**Figure 9:**
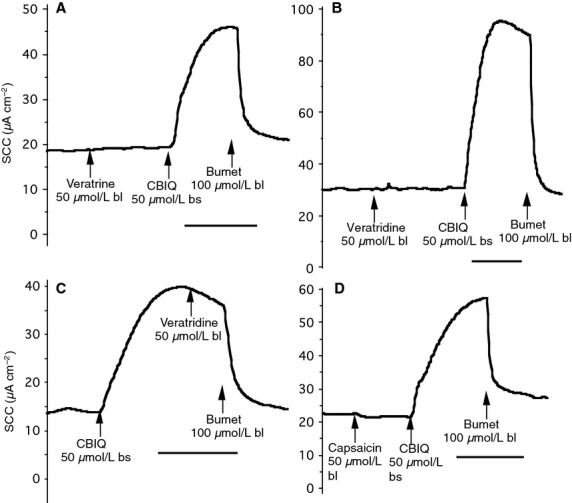
Lack of effect of veratrine and veratridine on SCC in Calu-3 cell monolayers. Neither veratrine or veratridine had any effect on basal SCC in Calu-3 cells (A and B). Similarly veratridine was without effect after the SCC had been increased by CBIQ (C), while the CBIQ response remained sensitive to bumetanide (bumet). Agents were added to either the basolateral (bl) or both sides (bs) of the Calu-3 monolayers. Similarly no effects of capsaicin were found (D). All the time bars are 500 sec.

While we have shown that the effects of the veratrine alkaloids on the submucosal glands appear to be neurally mediated there remains a possibility that voltage-sensitive sodium channels might exist on the glands cells themselves thus conferring sensitivity to veratrine and to TTX. To investigate this possibility, we have used Calu-3 cells, derived from the secretory epithelial cells of human submucosal glands (Shen et al. 1994). Calu-3 cells can be grown as epithelial monolayers capable of transporting salts and water under SCC conditions. Monolayers of Calu 3 epithelia, 1 cm^2^, were mounted in Ussing chambers and used to measure SCC. In all 11 experiments carried out, six were with veratrine and five with veratridine. In no experiment did the alkaloids have any effect on SCC whether added before or after anion secretion had been stimulated with CBIQ (Szkotak et al. 2004) that simultaneously activates CFTR chloride channels and KCNN4 potassium channels. This indicates that the model tissue contains no voltage-sensitive sodium channels. Assuming the model accurately portrays the properties of the secretory cells of airway glands it implies they are insensitive to veratrine alkaloids. In further experiments, it was shown that capsaicin too had no effect on SCC in Calu-3 monolayers. Some examples of these data are illustrated in Fig.9A–D.

## Discussion

A key feature of this study is the reliance upon veratrine alkaloids as activators of conducted action potentials in the neurones of the mucosal plexus in sheep airways. There is a vast literature (see Ulbricht 1969) that supports the view that the alkaloids selectively affect excitable membranes and more specifically voltage-sensitive sodium channels. We have employed both veratridine and veratrine in this work and have never found any qualitative difference between them. Veratrine consists mainly of veratridine with small amounts of other alkaloids with similar structure and function and derived from liliaceous species. Veratrine was used clinically as a counterirritant and in rheumatic conditions, but abandoned because of its toxicity to mucus membranes.

Activation of neurones in the mucosal plexus not only activated glandular secretion (Figs.1, 2) but also increased SCC (Fig.6). In the latter the effect was small (circa 10 *μ*A/cm^2^) compared with major effects that are obtained in similar experiments in epithelia from intestinal tissues (see Sheldon et al. 1990; Hyland and Cox 2005; O'Malley et al. 2012) where the enteric system is more densely expressed. Importantly both the effects on gland secretion and SCC were blocked by TTX. This finding consolidates the view that the alkaloids activate conducted action potentials in the mucosal plexus that are blocked when the voltage-gated sodium channels are inhibited by the toxin (Kao 1966). Glandular secretion can still be increased when neural activity is blocked by TTX by using agents acting directly on the glands, for example, in Fig.3 it is shown that veratrine has no effect when TTX is present, whereas forskolin acting directly on the glands stimulates brisk glandular secretion by activating the adenylate cyclase cascade (Joo et al. 2009).

In a further series of experiments (Fig.4A–F) we showed that muscarinic M3 receptor agonist, carbachol, can stimulate gland secretion in the presence of TTX. However, when this directly acting agonist is inhibited by the muscarinic antagonist atropine gland secretion can still be induced by forskolin. Taken together the data in Figs.3, 4 support the contention that traffic in the intrinsic nerve net of the trachea can cause secretion from the submucosal glands as well as causing an increase in SCC. It is beyond the scope of this study to investigate the nature of the transmitters involved, but investigations by others makes it likely that substance P and vasoactive intestinal polypeptide are strong contenders (Khansaheb et al. 2011).

Returning to the SCC response to veratrine (Fig.6) it seems probable that it was due to anion secretion as it occurred in the presence of amiloride, sufficient to block all sodium absorption, and could be mimicked by forskolin, demonstrated to cause anion secretion in the sheep trachea (Joo et al. 2009).

It can be noticed from Figs.4 that tracheal submucosal glands show low level gland secretion before any other secretion modifying agent is added. To determine if this results from tonic activity in the nerve net or is due to inherent activity in the glands themselves we used TTX. We showed that basal secretion rate did not alter during 50 min and further neither was it modified by addition of TTX to block any tonic activity in the neurones (Fig.5). This finding forces the view that one component of the basal secretion at least originates in the glands themselves. Therefore, it is arguable that in vivo both a neurally mediated and glandular component are the basis of the background secretion from the glands. In a recent study, Baniak et al. (2012) have shown that two cytokines (interleukin-1*β* and tumor necrosis factor-*α*) cause a cAMP-dependent increase in fluid secretion in pig airway submucosal glands. These cytokines are produced in response to Pseudomonas aeruginosa infection. The effects of the cytokines are relatively small (100 pL/min) compared to the rates shown in this study but fit the sentinel duties of a vigilant innate defense system. It would be of interest to know if the effects seen by Baniak were inhibited by TTX.

In the search for a stimulus that might trigger the innate defense response in sheep airways, we followed the lead of others (Khansaheb et al. 2011) and used the noxious stimulus capsaicin to activate the TRPV1 receptor (Szallasi and Blumberg 1999). Even after many attempts we failed to see any glandular response in the sheep although mice, pig, and human airways had shown responsiveness, but in general these species were more responsive to a variety of stimuli compared to the sheep (Joo et al. 2009). However, even in the responding species the maximal responses were small, approximately 0.4 nL/min per gland or less, especially when compared with the secretion rates produced by veratrine alkaloids or drugs that activate the adenylate cyclase cascade.

In spite of failing to see any glandular secretion in response to capsaicin we showed that capsaicin caused an immediate SCC response that rapidly desensitized, indicating that one aspect the capsaicin receptor apparatus was intact when recording in the SCC mode.

As we know that veratrine affects SCC (Fig.6) it can be concluded that some fibers of the mucosal plexus must terminate close to the apical surface (Dey et al. 1999). We were curious about the consequences of this process of secretion from the apical surface if the measurements were made in the configuration used for quantifying glandular secretion. Transport of salts across the apical membrane requires the obligatory movement of water to maintain osmotic balance and maybe the cause of so-called ‘surface secretion’ (Wine et al. 2011). As we show in Fig.7 veratrine apparently increases the rate of accumulation of surface secretion where the fates of 14 tiny bubbles were followed for over 1 h, each bubble contributed approximately 3.8 pL/min before veratrine and 37 pL/min afterward. If this hypothesis for the basis of surface secretion is correct it emphasizes the small contribution made by the surface epithelium to total airway surface secretion compared with that from the glands, at least in major airways (Trout et al. 2001).

While nerve cells and skeletal muscle cells have well characterized, veratridine-sensitive sodium ion channels that are readily blocked by TTX. TTX-sensitive sodium channels have also been shown to be present in unexpected places such as adrenal gland cells (Kirkepar and Prat 1979), fibroblasts (Munson et al. 1979), glioma cells (Reiser and Hamprecht 1983), and carotid body cells (Sato et al. 1989). This meant we had to consider the possibility that the secretory cells of the airway submucosal glands is the locus for the action of both the veratrine alkaloids and the antagonism by TTX. Calu-3 cells are as close as it is possible to get at the present time to a pure line of airway secretory cells (Shen et al. 1994). Fortunately they grow well on Millipore filters to form epithelial monolayers that generate CFTR-dependent chloride secretory responses in response to appropriate challenge, such as with forskolin. We found that Calu-3 monolayers were completely insensitive to either the alkaloids or to capsaicin, supporting the view that the effects described in this study were indirect and were neurally mediated.

In conclusion, we have shown that the veratrine alkaloids acting on neural elements in the mucosa of the ovine trachea cause a profuse secretion from the submucosal glands accompanied by a modest increase in anion secretion from the apical epithelial surface. These effects are abolished by TTX.

We have shown that low level glandular secretion, insensitive to TTX, still occurs suggesting gland cells are capable of assembling small volumes of secretory product without neural input. Nevertheless, normal respiration will continue to bombard the lining of the airways and inevitably will contain unwanted infectious agents. Release of cytokines or bacterial expression of flagellin (Baniak et al. 2012; Illek et al. 2008) gives an increase in CFTR-dependent chloride secretion, such that focal responses summate with general background secretion to maintain mucociliary clearance.

In these instances, the formation of cytokines is maybe part of an innate immune response to bacterial infection. In cystic fibrosis, where CFTR-dependent chloride secretion is disabled, transplanted lungs retain the ability to mount a defense against the dangers of breathing.

## Conflict of Interest

None declared.
